# Humoral Immunity to Varicella Zoster Virus in Patients with Systemic Lupus Erythematosus and Rheumatoid Arthritis Compared to Healthy Controls

**DOI:** 10.3390/vaccines9040325

**Published:** 2021-04-01

**Authors:** Marco Krasselt, Christoph Baerwald, Uwe G. Liebert, Olga Seifert

**Affiliations:** 1 Rheumatology Unit, Clinic for Endocrinology, Nephrology and Rheumatology, Department of Internal Medicine, Neurology and Dermatology, University of Leipzig, Liebigstr. 20, 04103 Leipzig, Germany; christoph.baerwald@medizin.uni-leipzig.de (C.B.); olga.seifert@medizin.uni-leipzig.de (O.S.); 2 Institute for Virology, University of Leipzig, Johannisallee 30, 04103 Leipzig, Germany; liebert@medizin.uni-leipzig.de

**Keywords:** varicella zoster virus, VZV antibodies, shingles, herpes zoster, systemic lupus erythematosus, SLE, rheumatoid arthritis, RA

## Abstract

Background: The prevalence of herpes zoster (HZ) is high in patients with rheumatic diseases. Systemic lupus erythematosus (SLE) doubles the risk for developing HZ. However, little is known about natural humoral immunity against varicella zoster virus (VZV) in patients with SLE. Hence, we compared VZV IgG antibody concentrations in a group of SLE patients with healthy controls and patients with rheumatoid arthritis (RA). Methods: *n* = 56 patients with SLE, *n* = 54 patients with RA, and *n* = 56 healthy controls were included in this study. The VZV IgG antibody concentration was measured using an enzyme-linked immunosorbent assay (ELISA). The antibody concentrations were compared between the groups. Results: Overall IgG antibody titers for VZV in SLE patients were comparable to healthy controls but higher when compared to patients with rheumatoid arthritis (*p* = 0.0012). In consequence, antibody levels in controls were higher than in RA patients (*p* = 0.0097). Stratification by age revealed highest titers among SLE patients in the fourth life decade (*p* = 0.03 for controls, *p* = 0.0008 for RA patients) whereas RA patients in their sixth decade had the lowest antibody concentration (*p* = 0.03 for controls, *p* = 0.04 for SLE patients). Regarding the individual HZ history, antibody levels of SLE patients with a positive history exceeded all other groups. Conclusions: Although humoral VZV immunity in SLE patients is comparable to healthy controls it seems to be pronounced in young SLE patients between 30 and 39. The lowest VZV IgG levels were found in RA patients. HZ seems to induce antibody production, particularly in patients with SLE. Immunological processes might contribute to VZV antibody levels in SLE patients, but further investigations are needed to substantiate this hypothesis. Even though the increased HZ prevalence seems to be independent of humoral immunity in SLE patients, reduced humoral immunity might contribute to HZ in RA patients. The available HZ subunit vaccination might be an appropriate way to reduce the HZ risk in patients with rheumatic diseases.

## 1. Introduction

With a lifetime risk of 20–30% in general and up to 50% in people reaching 85 years of age [1], herpes zoster (HZ, shingles) is a relevant clinical problem. Current investigations show an overall incidence of 9.9/1.000 person-years [2]. HZ is the result of an endogenous reactivation of a latent varicella zoster virus (VZV) infection, persisting in the sensory ganglia [3]. The most important complication of HZ is postherpetic neuralgia (PHN), a neuropathic pain syndrome that can persist for years and is often intractable [3]. The relevance of HZ is particularly high in patients with inflammatory rheumatic diseases. Within this large group, patients with systemic lupus erythematosus (SLE, relative risk (RR) up to 4.11) and rheumatoid arthritis (RA, RR up to 2.4) show a particularly increased risk for acquiring HZ [4,5,6]. This increased risk leads to high incidences of up to 91/1.000 person-years in SLE and 12/1.000 person-years in RA patients [5,7]. To date, two different vaccines have been approved to prevent HZ: a live-attenuated vaccine in 2006 and a subunit vaccine in 2017/2018. Increasing use of the available vaccines might reduce the striking incidence in inflammatory rheumatic diseases in the future. Nevertheless, the knowledge concerning humoral VZV immunity in SLE and RA patients is limited. We therefore compared VZV IgG antibodies in patients with SLE to both RA patients and controls.

## 2. Methods and Study Design

Consecutive patients with SLE or RA (≥18 years), matched for age and sex, were recruited from our outpatient clinic for participation in this study. SLE patients fulfilled the American College of Rheumatology (ACR) criteria for SLE [8], and RA patients the 2010 criteria of the ACR/European League Against Rheumatism (EULAR) [9]. Herpes zoster history was taken during the visit or was available from the existing medical records.

To compare the VZV antibody titers, controls without any history of inflammatory rheumatic or other relevant autoimmune diseases were randomly selected from the University Hospital of Leipzig and were age- and sex-matched. VZV IgG antibody concentrations were measured with an IgG enzyme-linked immunosorbent assay (ELISA) employing highly purified whole virus lysate strain Ellen.

The ethics committee of the University of Leipzig approved the design of the study, and written informed consent was obtained from each participant before study enrollment.

### Biostatistical Analysis

To report continuous data in this study, mean and standard deviation were used. Categorical data were described by absolute or relative frequencies. Fisher’s exact test was performed to compare frequencies of categorical variables. For the comparison of continuous data, either student’s t test or the Mann–Whitney U test was used after performing the Kolmogorov–Smirnov normality test. Holm-Šidák’s correction for multiple comparisons was applied for the subgroup analysis. Correlation between two parameters was analyzed with either Pearson’s product–moment correlation or Spearman, depending on the data distribution. A significant statistical difference was assumed when the *p*-value was below 0.05. Statistical analysis and figure preparation was conducted using GraphPad PRISM Version 8 for Mac (GraphPad Software Inc., San Diego, CA, USA).

## 3. Results

In total, 56 SLE (mean age 49.8 ± 15.9 years) and 54 RA patients (mean age 51.3 ± 13.4 years) were enrolled in this study. Furthermore, 56 controls (mean age 51.9 ± 14.8 years) were randomly selected from the diagnostic in-house routine. None of the participants had an HZ vaccination. There were no significant differences in age and sex between the patient groups and the healthy control group.

Details on the included patients and controls can be obtained from Table 1, and the medication of the patients is presented in Table 2.

### 3.1. VZV IgG Concentrations in SLE Patients Are Comparable to Healthy Controls but Higher Than in RA Patients

**Figure 1:**
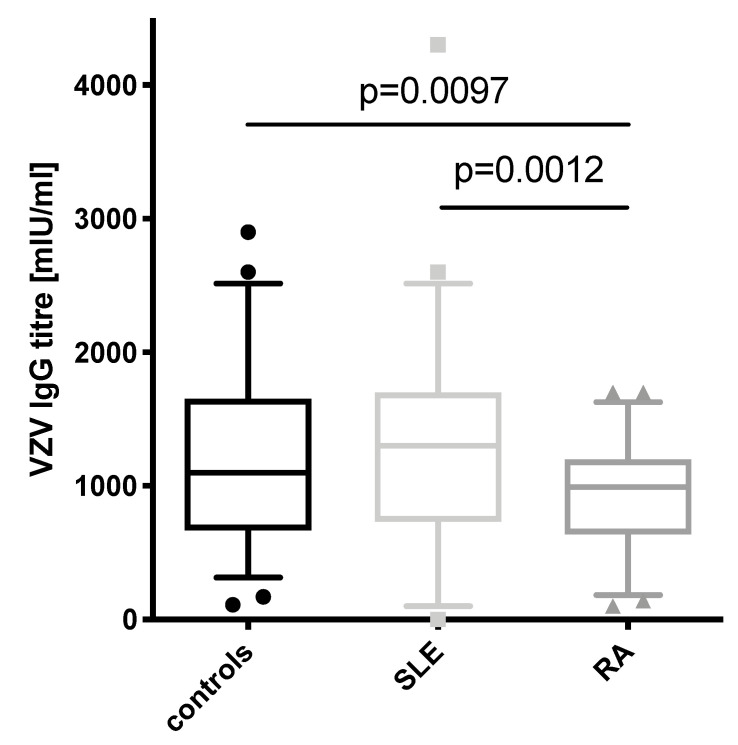
Varicella zoster virus (VZV) IgG antibody concentration among the three groups (healthy controls—black bar, SLE—light grey bar, RA—dark grey bar). Boxplots depict median with whiskers from the 5th to 95th percentile.

When comparing the VZV IgG levels between the three groups (Figure 1), the levels in SLE patients were comparable to controls but significantly higher than in RA patients (*p* = 0.0012). Furthermore, titers were higher in controls than in RA patients (*p* = 0.0097). No correlation between the individual medication and antibody levels was found within the two patient groups. Looking at all patients (SLE and RA), though, the use of azathioprine was positively correlated with the antibody titer (r = 0.274, *p* = 0.004). The meaning of this finding is limited by the fact that only one RA patient was receiving azathioprine.

### 3.2. Young SLE Patients Show the Highest VZV Titers

**Figure 2:**
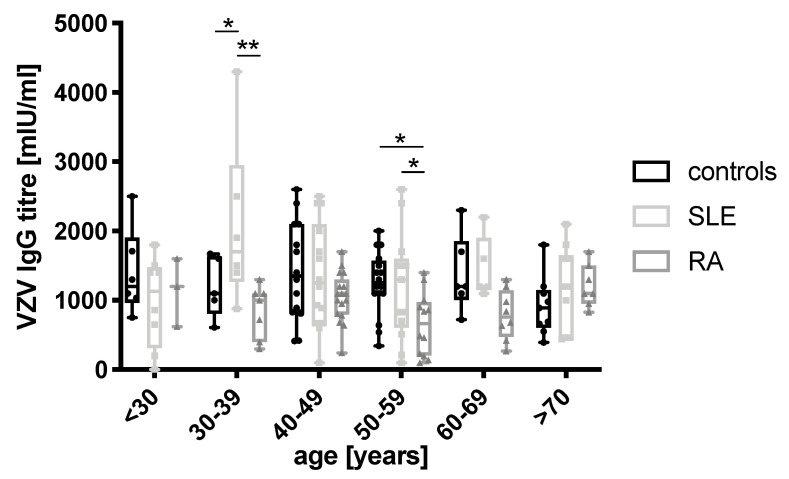
VZV antibody concentration stratified by age (healthy controls—black bars, SLE—light grey bars, RA—dark grey bars). Boxplots show median and whiskers from minimum to maximum. Additionally, all individual values (each symbol represents one participant) are superimposed to illustrate the size of each group. ** *p* < 0.01; * *p* < 0.05.

A stratification by age revealed the VZV IgG titers to be highest in SLE patients between 30 and 39 years (*n* = 6) compared to both controls (*n* = 5, *p* = 0.03) and RA patients (*n* = 7, *p* = 0.0008). Interestingly, RA patients between 50 and 59 years (*n* = 12) had significantly lower levels than the controls (*n* = 16, *p* = 0.03) and SLE patients (*n* = 13, *p* = 0.04). Beyond the age of 60, no difference in VZV IgG levels was found throughout the three groups (Figure 2).

### 3.3. History of Herpes Zoster in SLE Patients Is Associated with Higher Antibody Levels

**Figure 3:**
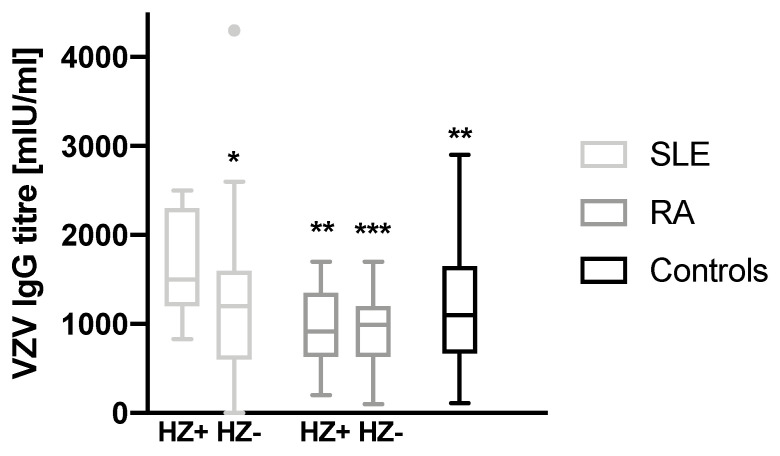
Comparison of VZV antibody titers between the three groups (SLE—light grey bars, *n* = 17 vs. 39; RA—dark grey bars, *n* = 14 vs. 40; controls—black bars, *n* = 56) after stratification by history of herpes zoster. Please note that there is no HZ history available for the controls. All depicted significances are related to SLE patients with positive HZ history (first light grey bar). No other inter-group differences were found. The boxplots are depicted using median and Tukey’s method. *** *p* < 0.001; ** *p* < 0.01; * *p* < 0.05.

The mean age at developing HZ was 33.5 ± 12.4 years (SLE) and 36.7 ± 12.4 years (RA) in our cohort. The HZ frequency was comparable in both patient groups (30.4 vs. 25.9% in SLE and RA patients, respectively). The mean time from onset of the rheumatic disease to HZ was shorter in SLE patients (6.8 ± 5.9 vs. 15.2 ± 10.2 years, *p* = 0.04, Table 1). When comparing the VZV IgG levels between SLE patients with positive HZ history and any other group (including SLE patients without HZ history, but also RA patients and controls), SLE patients show higher titers after at least one episode of HZ (Figure 3*,* statistical significances as indicated). No other inter-group differences were found in that regard. HZ history was not available for the controls. Furthermore, in both patient groups, no correlation between the age at HZ and the antibody level was revealed. Among SLE patients, prednisolone use (regardless the dose) was associated with a higher risk for HZ (*p* = 0.01).

## 4. Discussion

The aim of this study was to compare humoral immunity against VZV in SLE patients to patients with RA and healthy controls. Patients with inflammatory rheumatic diseases, in particular with SLE and RA, have an increased risk of developing herpes zoster (HZ) during their lifetime [4,5,6,10,11]. Age, use of immunosuppressives (including small molecules such as baricitinib), and glucocorticoid therapy increase that risk further [12,13,14]. In our cohort, an association between HZ and glucocorticoid use was evident in SLE patients only.

Both SLE and RA have a different and complex pathophysiology. SLE is often referred to as the prototype autoimmune disease with B-/T-cell exaggeration, autoantibody production, and immune complex deposition. RA, however, involves synovitis with infiltration of mononuclear cells and disease perpetuation is driven by cytokines such as tumor necrosis factor (TNF) [15].

The knowledge on immunity against VZV in inflammatory rheumatic diseases is scarce, mostly focusing on cellular rather than humoral immunity [7,16,17,18,19]. Of particular interest is our observation that young SLE patients in their fourth decade of life harbored higher titers than both controls and RA patients. Other researchers found higher overall titers of VZV IgG in SLE patients (*n* = 38) compared to healthy controls, independent of their age [7]. The authors speculated that SLE flares could be responsible for subclinical virus reactivations and therefore lead to increased VZV antibody levels. Of importance, no individual HZ history was available in that study [7]. A further investigation showed no connection between SLE disease activity and VZV antibody levels [20], making increased VZV titers due to subclinical reactivations in SLE flares unlikely [7,20]. Our results lead to the assumption that at least one episode of HZ is related to higher VZV titers in SLE patients. This finding is in line with the report of Nagasawa et al., who measured antibodies using a neutralization test [21]. Polyclonal hypergammaglobulinemia is frequently seen in patients with SLE [16,22,23,24], most likely due to B-cell activation [25]. Therefore, polyclonal IgG elevation was believed to be a possible explanation for high VZV IgG levels in SLE patients [21,26]. This assumption was questioned when IgG antibodies against diphtheria were found to be lower in SLE patients than in age-matched healthy controls, while VZV IgG antibodies were increased in the same SLE cohort [7]. Hypergammaglobulinemia as a consequence of polyclonal B-cell activation is also known in RA patients [24,27,28]. Antibodies against VZV, however, were lowest in RA patients in our cohort. Furthermore, we previously demonstrated that VZV antibody avidity is decreased in RA patients compared to matched controls [29]. Reactivation of VZV (either with HZ disease or subclinical) is thought to stimulate production of VZV IgG antibodies [30], a process called endogenous boosting [31]. However, VZV IgG levels in SLE patients with a positive HZ history not only exceeded those in SLE patients without a HZ history, but also those in RA patients regardless of their HZ history. Furthermore, antibody levels of SLE patients with a positive history were also higher than in controls. Although these findings emphasize the impact of a prior HZ on the VZV antibody levels in SLE patients, they also imply the existence of SLE-specific reasons. In a recent trial, combined therapy targeting B-cells with rituximab (CD20 antibody) and belimumab (B-cell activating factor (BAFF) antibody) in SLE patients led to a significant decrease of anti-VZV IgG, whereas both anti-tetanus IgG and anti-rubella IgG remained stable [32]. The authors speculated that autoreactive B-cells might have an increased BAFF dependence due to the continuous (auto-)antigen presence. In our cohort, only one SLE patient was under rituximab therapy, not allowing us to draw meaningful conclusions in that regard. Nevertheless, this speculation could explain the impact on VZV antibodies since VZV persists in the cranial nerve and dorsal root sensory ganglia after primary infection [32,33]. Indeed, high BAFF levels result in an enhanced humoral immunity and are associated with an increased SLE risk [34]. Therefore, the high levels of VZV antibodies in SLE patients might be the combined consequence of both autoreactive B-cells and prior HZ.

Despite the increased VZV antibodies, HZ incidence was high in SLE patients. The elevated VZV levels in the SLE patients in our cohort in their fourth decade of life might also be explained by the peak frequency of HZ at this age (mean age 33.5 ± 12.4 years). RA patients in our cohort had a similar mean age when developing HZ (36.7 ± 12.4 years), but lower antibody levels, emphasizing the suggested SLE-related reasons discussed above. An explanation for the particularly reduced antibody concentrations among RA patients in their sixth decade, probably hampering VZV immunity, might be premature immunosenescence, as discussed elsewhere [29,35].

During the last years, evidence accumulated that cell-mediated rather than humoral immunity is likely to be of utmost importance for controlling VZV reactivation. Cell-mediated immunity (measured using the VZV skin test) inversely correlates with both the severity of HZ skin lesions and pain [36,37]. In this context, the incidence of PHN might be associated with the decline of VZV-specific cell-mediated immunity [38]. However, humoral immunity, i.e., an antibody level increase, correlates with vaccine efficacy [39]. We previously showed that humoral VZV immunity seems to be reduced in RA patients [29]. The results at hand confirm this finding. Unfortunately, HZ vaccination indication in patients with rheumatic diseases remain unclear and reported vaccination rates are generally low [40,41,42,43,44]. The EULAR recommends HZ vaccination in patients with inflammatory rheumatic diseases “at high risk” only [45], and the ACR recommends a vaccination (live vaccine) for RA patients under treatment with disease-modifying antirheumatic drugs excluding any biologic therapy [46]. German vaccination guidelines, however, recommend HZ vaccination using the subunit vaccine for all patients with SLE or RA ≥ 50 years [47]. This recommendation is based on the pivotal subunit vaccine trials [48,49] and investigations on the efficacy and safety in other immunocompromised patient groups (e.g., patients after peripheral stem cell transplantation or renal transplant recipients) [50,51,52,53,54]. Although the live vaccine was shown to be effective and well tolerated in stable SLE patients in a randomized trial [55] and retrospective analyses also indicated a reduced incidence of HZ for other individuals under immunosuppressive medication, including RA patients [56,57], its effectiveness is limited and reduced in the elderly (<70 years 63.9%, ≥70 years 37.6%) [3]. The subunit vaccine, however, demonstrated a high efficacy of 97.2 and 89.8%, respectively (patients ≥ 50 and ≥70 years) [48,49]. Recently published post-marketing safety data are promising [58,59]. Data on effectiveness and safety in patients with inflammatory rheumatic diseases, however, are still scarce, except for preliminary data focusing on RA patients [60,61].

Our study has several limitations. Most importantly, no HZ history was available from the controls, limiting the subgroup analysis to both of the patient groups. On the other hand, we were able to compare the four subgroups of the two rheumatic entities to a healthy control group, gaining insight into humoral immunity of both SLE and RA patients. In addition, the sample size is relatively small, but comparable to other investigations in that context. Nevertheless, we believe that our results may contribute to furthering our understanding of VZV immunity, particularly in patients with rheumatic diseases.

## 5. Conclusions

HZ is a relevant clinical problem in patients with inflammatory rheumatic diseases. Incidence is highest in SLE patients despite increased antibody levels in young patients. Clinicians should therefore not rely on high antibody levels, suggesting proper immunity. Besides prior HZ episodes, BAFF might be involved in the seemingly adequate humoral immunity in SLE patients, but clearly further studies including cell-mediated VZV immunity are needed to underline this speculation. Vaccination against HZ should be considered in patients with rheumatic diseases, particularly in SLE and RA patients, who are both at a high risk.

## Figures and Tables

**Table 1:** Epidemiology of patients and controls. Given are mean and standard deviation (SD).

	SLE Patients *N* = 56	RA Patients *N* = 54	Controls *N* = 56
**Mean age, years**	49.8 ± 15.9	51.3 ± 13.4	51.9 ± 14.8
**Female, *n* (%)**	54 (96.4)	47 (87)	53 (94.6)
**Subjects with positive HZ history, *n* (%)**	17 (30.4)	14 (25.9)	n.a.
**Mean age at HZ, years**	33.5 ± 12.4	36.7 ± 12.4	n.a.
**Mean time from disease onset to HZ, years**	6.8 ± 5.9	15.2 ± 10.2	n.a.

**Table 2:** Medication of the studied patients with SLE and RA (*n* = 56 and 54, respectively).

Characteristics	SLE	RA
*Medication, n (%)*		
All immunosuppressives	38 (67.9)	50 (92.6)
Prednisolone	44 (78.6)	44 (81.5)
Hydroxychloroquine	13 (23.2)	0 (0)
Azathioprine	18 (32.1)	1 (1.9)
Mycofenolat-mofetil	17 (30.4)	0 (0)
Cyclophosphamide	1 (1.8)	0 (0)
Methotrexate	1 (1.8)	33 (61.1)
Leflunomide	0 (0)	5 (9.3)
Sulfasalazine	0 (0)	1 (1.9)
*bDMARDs, n (%)*		
Adalimumab	0 (0)	12 (22.2)
Etanercept	0 (0)	6 (11.1)
Abatacept	0 (0)	4 (7.4)
Rituximab	1 (1.8)	2 (3.7)
Certolizumab	0 (0)	2 (3.7)
*tsDMARDs, n (%)*		
Tofacitinib	0 (0)	2 (3.7)

Given are numbers (%). bDMARD—biological disease-modifying antirheumatic drug; tsDMARD—targeted synthetic disease-modifying antirheumatic drug.

## Data Availability

The data presented in this study are available on reasonable request from the corresponding author. The data are not publicly available due to privacy reasons.
